# Heat-transfer performance of twisted tubes for highly viscous food waste slurry from biogas plants

**DOI:** 10.1186/s13068-022-02156-4

**Published:** 2022-07-06

**Authors:** Jingjing Chen, Mikael Risberg, Lars Westerlund, Urban Jansson, Changsong Wang, Xiaohua Lu, Xiaoyan Ji

**Affiliations:** 1 Energy Engineering, Division of Energy Science, Luleå University of Technology, 97187 Luleå, Sweden; 2 State Key Laboratory of Material-Oriented Chemical Engineering, Nanjing Tech University, 210009 Nanjing, People’s Republic of China; 3 Boden Biogas Plant, Smidesvägen 3, 96138 Boden, Sweden

**Keywords:** Food waste slurry, Rheological properties, Twisted tubes, Computational fluid dynamics, Heat-transfer enhancement

## Abstract

**Background:**

The use of food waste as feedstock shows high production of biogas via anaerobic digestion, but requires efficient heat transfer in food waste slurry at heating and cooling processes. The lack of rheological properties hampered the research on the heat-transfer process for food waste slurry. Referentially, the twisted hexagonal and elliptical rubes have been proved as the optimal enhanced geometry for heat transfer of medium viscous slurries with non-Newtonian behavior and Newtonian fluids, respectively. It remains unknown whether improvements can be achieved by using twisted geometries in combination with food waste slurry in processes including heating and cooling.

**Results:**

Food waste slurry was observed to exhibit highly viscous, significant temperature-dependence, and strongly shear-thinning rheological characteristics. Experiments confirmed the heat-transfer enhancement of twisted hexagonal tubes for food waste slurry and validated the computational fluid dynamics-based simulations with an average deviation of 14.2%. Twisted hexagonal tubes were observed to be more effective at low-temperature differences and possess an enhancement factor of up to 2.75; while twisted elliptical tubes only exhibited limited heat-transfer enhancement at high Reynolds numbers. The heat-transfer enhancement achieved by twisted hexagonal tubes was attributed to the low dynamic viscosity in the boundary layer induced by the strong and continuous shear effect near the walls of the tube.

**Conclusions:**

This study determined the rheological properties of food waste slurry, confirmed the heat-transfer enhancement of the twisted hexagonal tubes experimentally and numerically, and revealed the mechanism of heat-transfer enhancement based on shear rate distributions.

**Supplementary Information:**

The online version contains supplementary material available at 10.1186/s13068-022-02156-4.

## Highlights

- Rheological properties of food waste slurry were tested and modeled
- Pilot test of twisted tube heat exchanger validated the CFD-based simulations
- Twisted hexagonal tubes exhibited better performance at low-temperature differences
- Heat-transfer enhancement was due to strong and continuous shear effect close to tube walls

Twisted hexagonal tubes exhibited better performance at low-temperature differences

Heat-transfer enhancement was due to strong and continuous shear effect close to tube walls

**Supplementary Information:**

The online version contains supplementary material available at 10.1186/s13068-022-02156-4.

## Background

Food waste, a type of typical organic solid waste, is produced in large quantities and can severely contaminate air, water, and soil if it is not collected, transported, and stored properly [1]. Fortunately, food waste is degradable and is used as a desirable substrate in the production of biogas via the anaerobic digestion (AD) process [2–4]. Mohsen et al. [5] designed a laboratory-scale semi-continuous membrane-assisted anaerobic reactor to produce biogas from food waste slurry (FWS) with a total solid (TS) content of 13%. They found that a considerable amount of biogas can be produced while consuming extremely low amounts of chemicals, such as alkalis and active carbon. Charles et al. [3] investigated FWS with TS = 10% in a pilot-scale 900 m^3^ AD reactor and reported a high production rate of 600 m^3^ of biogas (60% of methane) per ton of volatile solids (i.e., the flammable part of solids at 550 °C). In practice, food waste has emerged as a vital feedstock in the AD process in biogas plants in several European countries, including Ireland [6], Denmark [7], and Sweden [8].

Usually, the thermal energy used to maintain the temperature of AD reactors and preheat feed streams accounts for more than 70% of the total energy utilization in a biogas plant [9]. Especially when food waste is used as the substrate, sanitation at a high temperature (70 °C) is required to pasteurize the feeding slurries [10], leading to the consumption of additional thermal energy compared to processes based on other substrates. Moreover, following sanitation, a cooling process is required to decrease the temperature from 70 to 50 °C to fulfill the temperature requirements of AD reactors. This makes the use of highly efficient thermal systems essential to reduce thermal energy utilization. The heat exchanger is an important component in such systems. Although, currently, shell-and-tube heat exchangers with circular tubes are the most popular variant, developing novel heat exchangers to improve the efficiency of thermal systems is one of the most important topics of research in this field.

For developing novel heat exchangers, the properties of the working fluids, especially the rheological properties for non-Newtonian fluids, should be determined first. The main working fluids in the AD process are the slurries. Manure and corn straw slurries have been found to be the non-Newtonian fluids and show complex properties for their shear-thinning, temperature-, and substrate-dependent rheological properties [11, 12]. Moreover, the rheological properties were found as the key to determining the flow and heat-transfer process for the slurries [13]. However, to the best of our knowledge, the rheological properties of food waste slurry are missing. This hindered the quantifications of experimental and numerical studies on the heat-transfer process for the food waste slurries.

The twisted tubes have been extensively approved as an effective passive technology for the heat-transfer enhancement of fluids with complex properties. In our previous studies, for the manure slurry (MS) [14] and corn straw slurry (CSS) [15], we screened twisted hexagonal tubes (THTs) from multiple twisted equilateral-polygon tubes using computational fluid dynamics (CFD)-based simulations, verified the enhanced performance of THTs with experiments, and integrated THTs into thermal systems of biogas plants. Our observations indicated that for the full-scale biogas plant, the implementation of optimally structured THTs in waste-heat recovery and the external heating processes significantly increase the net production of biofuel by up to 17% and conserve thermal energy by up to 39%, respectively. However, these studies focused on the heat-transfer performance including the heat-transfer coefficient, friction coefficient, and enhancement factor only compared to the circular tube in heating process (cool slurries and hot heat-exchange wall). The improvements achieved using THTs and commercialized twisted tubes in combination with other kinds of slurries, e.g., food waste, and in other processes, including heating and cooling, are yet to be established.

Twisted elliptical tubes (TETs) have been identified to be one of the most successful types of commercial heat exchangers in water treatment and chemical industries, and its enhancement factor (1.3–2.5) for simple fluids [16] is comparable to those of THTs for slurries. For slightly high viscous fluids, e.g., oil in the laminar region (0.030 Pa·s), significantly high enhancement factors up to 1.5 were obtained [17]; for engine oil (0.0033 Pa·s) and ethylene glycol (0.016 Pa·s), the enhancement factor was bounded by 0.94, and it was observed to decrease as the Reynolds number (*Re*) increased [18]. These results indicate that it might be worthwhile to extend the application of commercialized TET to slurries and compare its performance with that of the THT. However, to the best of our knowledge, this topic has not yet been researched.

In this study, the heat-transfer performances of FWS in THT and TET were evaluated, using the circular tube (CT) as the reference. To this end, the rheological properties of FWS with TS = 10% obtained from a biogas plant were tested and modeled. Based on these data, CFD-based simulations were conducted to predict the performance of heat exchangers with different geometries, and pilot-scale testing was performed to validate the numerical simulation results. Moreover, the performances of THT and TET in heating and cooling systems at different temperature differences were analyzed and compared to those of CT. Finally, the mechanism of heat-transfer enhancement was revealed.

## Results and discussion

In this section, the determination and modeling of the rheological properties of FWS are described, and their implementation in the CFD-based simulation of the heat-transfer process is discussed. Subsequently, the experimental data on the heat-transfer performance of FWS in THT obtained via pilot-scale testing are described. Further, the validations of CFD simulations are determined, and heat-transfer coefficients, friction coefficients during heating and cooling phases in THT and TET are determined using CFD and compared to those of CT. Thereafter, the enhancement factors of THT and TET are calculated based on the numerical results. Moreover, the relationships between operating conditions, flow state, and heat-transfer performance of FWS in THT are established with respect to practical applications. Finally, the mechanism of heat-transfer enhancement is revealed.

### Rheological properties of food waste slurry

#### Testing results

**Fig. 1:**
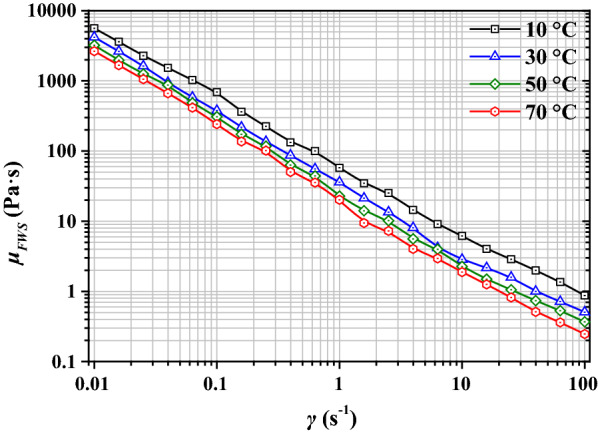
Rheological properties of food waste slurry (FWS)

The rheological properties for FWS with TS = 10% at temperatures between 10 °C and 70 °C and shear rates *γ* between 0.01 s^−1^ and 100 s^−1^ are listed in Additional file 1: Table S1 and depicted in Fig. 1. The dynamic viscosity of FWS is observed to be strongly dependent on temperature; it decreased by the factors of up to 2.2 at *γ* = 0.1 s^−1^ (*μ* = 5658 Pa·s at 10 °C *vs. μ* = 2612 Pa·s at 70 °C) and 3.5 at *γ* = 100 s^−1^ (*μ* = 0.8814 Pa·s at 10 °C *vs. μ* = 0.2487 Pa·s at 70 °C) when the temperature was increased from 10 to 70 °C. Shear rate is also observed to have a profound impact on the rheological properties of FWS. Low shear rates (*γ* = 0.1 s^−1^) induced ultra-high dynamic viscosities (*μ* = 263–543 Pa·s) in FWS, which is similar to that of polymers, such as polyphenylene sulfide [19].

#### Comparison of different slurries

A comparison of the rheological properties of different slurries is presented in Table 1. The viscosity of FWS was higher than those of MS and CSS by 1–2 orders of magnitude. However, at high shear rates (*γ* = 100 s^−1^), the dynamic viscosity of FWS was 2–3 times that of MS and one order of magnitude higher than that of CSS. The difference between the rheological properties of different slurries can be explained as follows. FWS and MS substrates comprise hydrophilic particles and soluble organics, while CSS is a typical lignocellulosic fiber suspension with hydrophobic behavior. In addition, FWS consists of larger organic molecules, e.g., starch, proteins, oils, and fats, compared to MS. Hence, the dynamic viscosity of FWS is higher than that of MS and much higher than that of CSS under identical shear conditions.

**Table 1:** Comparison of the dynamic viscosities of different slurries

Slurries	T, °C	*μ*, Pa·s	
		*γ* = 100 s^−1^	*γ* = 0.1 s^−1^
FWS, TS = 10% (this study)	10	0.72	543
	55	0.22	263
MS, TS = 10% [20]	10	0.32	38
	55	0.081	2.2
CSS, TS = 8% [15]	10	0.035	1.6
	55	0.032	0.75

#### Modeling

According to previous studies [21], slurries obtained from biogas plants are typical shear-thinning and highly viscous fluids. The power law model, given by Eq. (1), has been used to describe non-Newtonian behavior reliably in previous studies [12, 20]. Moreover, it is necessary to determine the critical-shear viscosity (CSV) and zero-shear viscosity (ZSV) of the rheology for application in the CFD-based simulations of the heat-transfer process of the slurries [22]. In this study, the tested rheological properties of FWS with TS = 10% were modeled using Eq. (1). The characteristic parameters, including the consistency coefficient, *k*, the flow behavior index, *n*, the CSV, *μ*_*CSV*_, and the ZSV, *μ*_*ZSV*_, were modeled using Eqs. (2), (3), (4), and (5), respectively. Considering the flattening tendency of *γ* in the range between 60 s^−1^ and 100 s^−1^, as depicted in Fig. 1, *μ*_CSV_ was selected to be equal to *μ* at *γ* = 100 s^−1^, and for the convergence of the simulation, *μ*_ZSV_ was selected to be equal to *μ* at *γ* = 0.01 s^−1^:

1 $$\mu = k\gamma^{n - 1} ,$$

2 $$k = 0.101e^{{\frac{1806.0}{T}}} ,R^{2} = 0.968,$$

3 $$n = - 0.00117T + \,0.358,R^{2} = 0.970,$$

4 $$\mu_{CSV} = 5.63 \times 10^{ - 4} e^{2072.8/T} ,R^{2} = 0.990,$$

5 $$\mu_{ZSV} = 74.4e^{{{{1224.0} \mathord{\left/ {\vphantom {{1224.0} T}} \right. \kern-\nulldelimiterspace} T}}} ,R^{2} = 0.973.$$

The characteristic parameters involved in the modeling of different slurries are listed in Table 2. For non-Newtonian fluids, a lower *n* represents stronger shear-thinning behavior. Thus, FWS slurry was observed to exhibit extremely strong shear-thinning behavior with the flow behavior, *n* being approximately equal to and less than zero at 10 °C and 55 °C, respectively. In comparison, the values of *n* for CSS and MS are intermediate between 0.1 and 0.519. This indicates that the dynamic viscosity of FWS is much more sensitive to the shear effect compared to those of CSS and MS. *k* is a measure of the average viscosity of non-Newtonian fluids. The value of *k* for FWS is higher than those of CSS and MS by more than one order of magnitude. This explains the much higher viscosity of FWS compared to those of CSS and MS.

**Table 2:** Rheological parameters of different slurries

Slurries	T, °C	*k*	*n*
FWS, TS = 10% (this study)	10	59.7	0.0409
	55	24.9	− 0.0239
MS, TS = 10% [20]	10	7.68	0.308
	55	0.739	0.519
CSS, TS = 8% [15]	10	1.57	0.178
	55	0.754	0.312

### Results of the pilot test

**Fig. 2:**
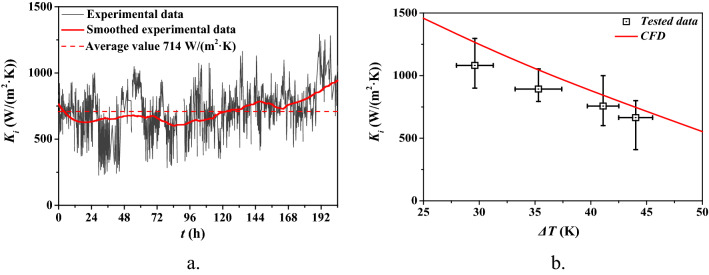
*K*_*i*_ of FWS in THT at 5 m^3^/h: **a** experimental values and **b** comparison of experimental data and simulated results

A continuous test (5–10 h per day; 204 h in aggregate; between 1st and 30th April, 2021) was conducted to determine the *K*_*i*_ of FWS with TS = 10% in THT heat exchangers at the Boden Biogas Plant, Sweden. The results of the pilot-scale experiment are illustrated in Fig. 2a. During the continuous test, obvious fluctuations were observed in the *K*_*i*_ values of the THTs. The flow rate of FWS was maintained at 5 m^3^/h, while the temperature of the stream of FWS varied owing to the operations. Hence, the fluctuations of *K*_*i*_ were induced by the unstable temperature differences. The average value of *K*_*i*_ is 714 W/(m^2^·K), which is 2.20 times that of CT heat exchangers (325 W/(m^2^·K) obtained in our previous tests [22]. The relationship between the *K*_*i*_ and the temperature difference, *ΔT*_*m*_, was extracted from Fig. 2a and is illustrated in Fig. 2b. The results confirmed that *K*_*i*_ of FWS in THT decreased with the increasing temperature difference.

### Heat-transfer performance of food waste slurry in twisted tubes

#### Validation

Before systematically evaluating the heat-transfer performance of FWS in twisted tubes, the numerical results were validated by simulating the *K*_*i*_ values of FWS with TS = 10% in THTs corresponding to temperature differences (*ΔT*, absolute value of *T*_*w*_–*T*_*s*_) ranging from 25 to 50 °C. The numerical CFD results, depicted in Fig. 5b, were validated by comparing them with the results obtained via pilot test. The average relative deviation (ARD) between the experimental and simulated results was 13.8%. Usually, ARDs between experimental and numerical results in the range of 10–15% represent reliable results in engineering [23]. Therefore, the CFD-based simulations are reliable.

#### Heat-transfer coefficients

**Fig. 3:**
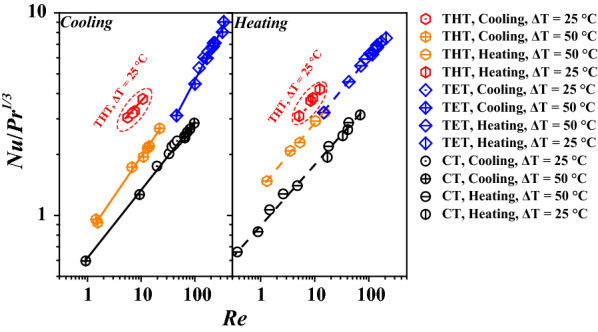
*K*_*i*_ values of FWS under different operating conditions in different tubes

Next, the heat-transfer coefficient *K*_*i*_ values of FWC in THT, TET, and TC during both heating and cooling processes [different wall and fluid-inlet temperatures (*T*_*w*_ and *T*_*s*_)] were estimated numerically. The *K*_*i*_ values corresponding to different temperature differences are depicted in Fig. 3. FWS was observed to exhibit significantly higher *Nu*/*Pr*^1/3^ in THT and TET than in CT at the same *Re*. Further, the *K*_*i*_ values of FWS in different tubes during heating and cooling phases were quite different.

The numerical results of *Nu* were also correlated with *Re* and *Pr*, as recorded in Table 3. The correlation of the *Nu* of FWS in THT exhibited the same tendency as that in TET corresponding to large *ΔT* = 50 °C [see Eqs. (3) and (5); (4) and (6) in Table 5]. Significantly, FWC exhibited better heat transfer in THT compared to TET at *ΔT* = 25 °C. *K*_*i*_ of FWC in TET and CT was observed to be hardly dependent on *ΔT*. This indicates that THT possesses an advantage in terms of heat exchange during various thermal processes, including heating, cooling, sanitation, and waste-heat recovery, in FWS-based biogas plants, over TET and CT at low-temperature differences.

**Table 3:** Correlations of *Re*, *Pr,* and *Nu* in different tubes

Tubes	Equations	
	Cooling	Heating
THT, *ΔT* = 25 °C	1. *Nu* = 1.69*Re*^*0.335*^*Pr*^*1/3*^, *R*^*2*^ = 0.973	2. *Nu* = 1.76*Re*^*0.346*^*Pr*^*1/3*^, *R*^*2*^ = 0.999
THT, *ΔT* = 50 °C	3. *Nu* = 0.798*Re*^*0.387*^*Pr*^*1/3*^, *R*^*2*^ = 0.993	4. *Nu* = 1.54*Re*^*0.265*^*Pr*^*1/3*^, *R*^*2*^ = 0.943
TET	5. *Nu* = 0.422*Re*^*0.523*^*Pr*^*1/3*^, *R*^*2*^ = 0.984	6. *Nu* = 1.36*Re*^*0.325*^*Pr*^*1/3*^, *R*^*2*^ = 0.996
CT	7. *Nu* = 0.617*Re*^*0.337*^*Pr*^*1/3*^, *R*^*2*^ = 0.995	8. *Nu* = 0.902*Re*^*0.295*^*Pr*^*1/3*^, *R*^*2*^ = 0.993

#### Friction coefficients

**Fig. 4:**
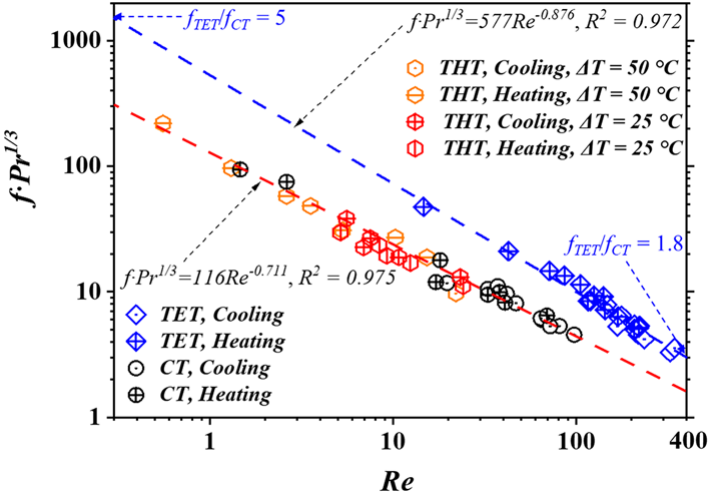
Friction coefficient *f* of FWS at different operating conditions in different tubes

The friction coefficient *f* should be considered during the application of an enhanced geometry to a heat-transfer process. Thus, the values of *f* for THT, TET, and CT corresponding to different values of *Re* and *Pr* were determined using CFD-based simulations. The results are illustrated in Fig. 4. The values of *f* of THT and CT exhibited similar trends (*f·Pr*^1/3^ = 116*Re*^−0.711^), while that of TET followed the equation, *f·Pr*^1/3^ = 577*Re*^−0.876^. This indicates that the flow resistance in THT is similar to that in CT and much lower than that in TET. For Newtonian fluids, the friction can be reportedly increased by up to 1.9 times in TET compared to that in CT (*f*_*TET*_/*f*_*CT*_ = 1.9) [24] in the laminar region, and a decrease in the relative value, *f*_*TET*_/*f*_*CT*_, is observed at Reynolds numbers ranging between 250 and 400 [25]. In the present study, for the highly viscous FWS, *f*_*TET*_/*f*_*CT*_ was observed to be 5–1.8 corresponding to *Re* lying in 0.3–400, indicating its advantage over TET in terms of the flow resistance, especially corresponding to low values of *Re*.

#### Enhancement factors

**Fig. 5:**
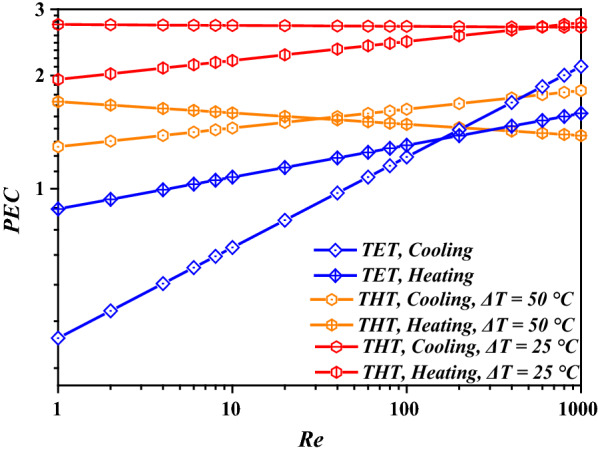
Enhancement factors of different twisted tubes corresponding to different temperature differences

In principle, the comprehensive enhancement factor—the performance evaluation criteria (*PEC*)—should be considered while designing enhanced geometries; the value of *PEC* is directly proportional to the quality of the heat-transfer performance, and *PEC* > 1.5 has been recommended for engineering applications [26]. The values of *PEC* of THT, TET, and CT were calculated based on the obtained *Nu* and *f*. The results are illustrated in Fig. 5. A considerable heat-transfer enhancement (with *PEC* up to 2.75) was achieved for FWS in THT with *ΔT* = 25 °C, while *PEC* was in the range of 1.2–1.8 for *ΔT* = 50 °C. Usually, *ΔT* decreases as the heat-transfer process progresses. For example, when the temperature of the working fluid is gradually increased by increasing the temperature of the walls of the tube, the transferred heat from the wall to the working fluid gradually decreases because the heat-transfer coefficient *Nu* changes slightly for normal heat exchangers (refer TET and CT in Fig. 3). Hence, THT is promising for practical applications of FWS in biogas plants for its increased *K*_*i*_ at low-temperature differences. Moreover, TET performs worse than even CT at *Re* < 5 and 50 for heating and cooling processes, respectively, while it exhibits relatively weak advantages over CT at *Re* > 400 compared to THT. Therefore, TET heat exchangers are not recommended for application in biogas plants handling FWS.

#### Engineering equation

**Fig. 6:**
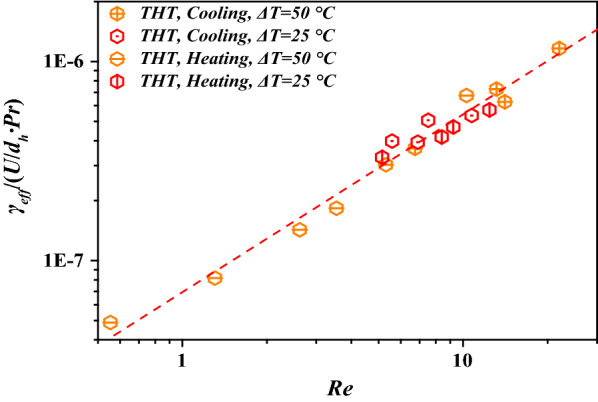
Effective shear rates at different operating conditions for THT

The effective shear rate *γ*_*eff*_ has been used to quantify the effective dynamic viscosity, *μ*_*eff*_ [14, 27]. The relationship between *γ*_*eff*_ and the operating conditions is displayed in Fig. 6 and regressed using Eq. (6). Using this relationship, *K*_*i*_ and *PEC* of FWS in THT can be determined directly from the tested properties and operating conditions. For engineering applications, *γ*_*eff*_ can be determined with Eq. (6). Subsequently, *μ*_*eff*_ can be calculated with Eqs. (1)–(3). Finally, the heat-transfer coefficient *K*_*i*_ can be calculated with Eqs. (7) and (8), the correlated Eqs. 1–4 in Table 3, and Eq. (9):

6 $$\gamma_{eff} = 7.09 \times 10^{ - 8} \frac{{\text{U}}}{{d_{h} }}{\text{Re}}^{0.881} Pr,R^{2} = 0.920,$$

7 $$Re = \frac{{d_{h} U_{\rho } }}{{\mu_{eff} }},$$

8 $$\Pr = \frac{{\mu_{eff} C_{p} }}{\lambda },$$

9 $$Nu = \frac{{K_{i} d_{h} }}{\lambda }.$$

### Mechanism of heat-transfer enhancement

For highly viscous FWS, THT exhibits a considerably high value of *K*_*i*_ with relatively low friction compared to TET, leading to superior enhancement compared to CT. Moreover, THT exhibits better heat transfer at lower temperature differences. To reveal the mechanism of these enhancements, it is essential to understand the dynamic viscosity and shearing state in tubes for FWS with non-Newtonian behavior.

In this part, firstly, the ratio of the dynamic viscosity in the boundary layer and bulk (*μ*_*bl*_*/μ*_*eff*_) and shear rate distributions in different tubes were sampled from the numerical results and analyzed. In our previous study [15], the average value of the shear rate *γ*_*avg*_ inside twisted can be used to represent the shear effect near the walls of the tubes. Hence, the dynamic viscosity in the boundary layer *μ*_*bl*_ was calculated based on *γ*_*avg*_ (refer to Additional file 1: Tables S2, S3, and S4 in supplementary material) obtained using CFD-based simulation following Eq. (4) and *μ*_*eff*_ was determined with Eq. (6). Subsequently, because the shearing state inside tubes determine the viscosity in tubes, the distributions of shear rate at different conditions were further analyzed.

#### Viscosities in boundary layer

**Fig. 7:**
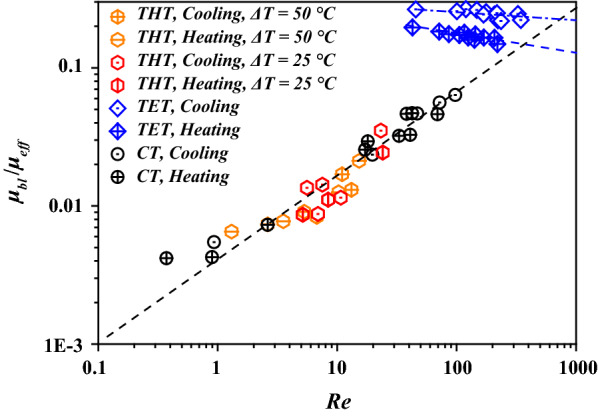
Dynamic viscosity of FWS in different tubes

The values of *μ*_*bl*_*/μ*_*eff*_ in different tubes are illustrated in Fig. 7. THT and CT exhibited similar trends in dynamic viscosity with respect to *Re*, while TET exhibited a much higher dynamic viscosity in the boundary layer. This explains the lower flow resistance in THT compared to that in TET. However, the trend of *μ*_*bl*_*/μ*_*eff*_ of THT indicates increased mixing, as represented by *Re*, and lower contribution to the reduction of the dynamic viscosity in the boundary layer compared to bulk flow. In TET, *μ*_*bl*_*/μ*_*eff*_ was observed to decrease with an increase in *Re*. Such dependence trends of *μ*_*bl*_*/*μ_eff_ on *R*e explain the heat-transfer enhancements achieved using THT and TET at low and high values of *Re*, respectively, as depicted in Fig. 5.

#### Shear rate distributions

**Fig. 8:**
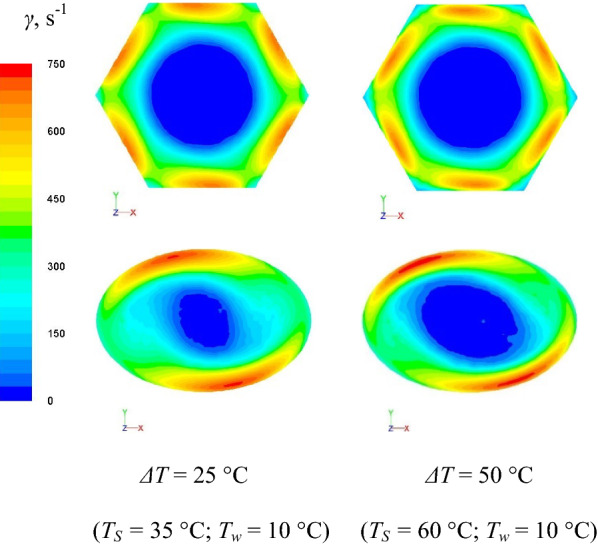
Shear rate distribution inside THT and TET at U = 2.5 m/s

Higher heat-transfer performance of FWS in THT was observed at lower temperature difference in experiments, as shown in Fig. 2b. However, the dynamic viscosities in the boundary layer in THT corresponding to varying values of *ΔT* were observed to exhibit the same tendency as that depicted in Fig. 7. A comparison of the shear rate distributions in THT and TET is depicted in Fig. 8. As established in our previous study [15], the strong and continuous shear effect near the walls of the tube leads to the strong and continuous local *K*_*i*_. The region near the walls in THT where the shear force was strong (*γ* > 600 s^−1^) was observed to be larger than its counterpart in TET. This explains the superior performance of THT compared to TET. Moreover, as shown in Fig. 8, although FWS exhibited a similar shear rate distribution at *ΔT* = 25 and 50 °C in THT, leading to the same tendency of *μ*_*bl*_*/μ*_*eff*_, it is apparent that the strong and continuous shear effect in the case of *ΔT* = 25 °C is constrained to a region that is closer to the walls than in the case of *ΔT* = 50 °C, leading to the lower-viscosity region close to the heat-exchange wall. This explains the higher degree of heat-transfer enhancement achieved using THT corresponding to low values of *ΔT*.

## Conclusions

This study determined the rheological properties of food waste slurry, confirmed the heat-transfer enhancement of the twisted hexagonal tubes experimentally and numerically, and revealed the mechanism of heat-transfer enhancement based on shear rate distributions.

FWS was observed to exhibit a strong temperature-dependence and an extremely high shear-thinning dynamic viscosity. The numerical method used in this study was verified to be reliable. Enhancement factors of up to 2.75 were achieved in THT at low-temperature differences. In contrast, TET exhibited a weakened performance compared to even CT at a low *Re* and limited enhancement at a high *Re*. Moreover, the cause of the heat-transfer enhancement in THT was identified as the strong and continuous shear rate close to wall.

The methodologies proposed in this study are expected to be useful as a reference for future studies on heat-transfer characteristics of non-Newtonian fluids with high dynamic viscosities.

## Materials and methods

In this section, the methodology used to test and model the rheology of FWS are described. Details of the numerical models used to determine the heat-transfer performance of THT, TET, and CT as well as those of the pilot-scale testing in a biogas plant are presented.

### Rheological properties of food waste slurry

**Fig. 9:**
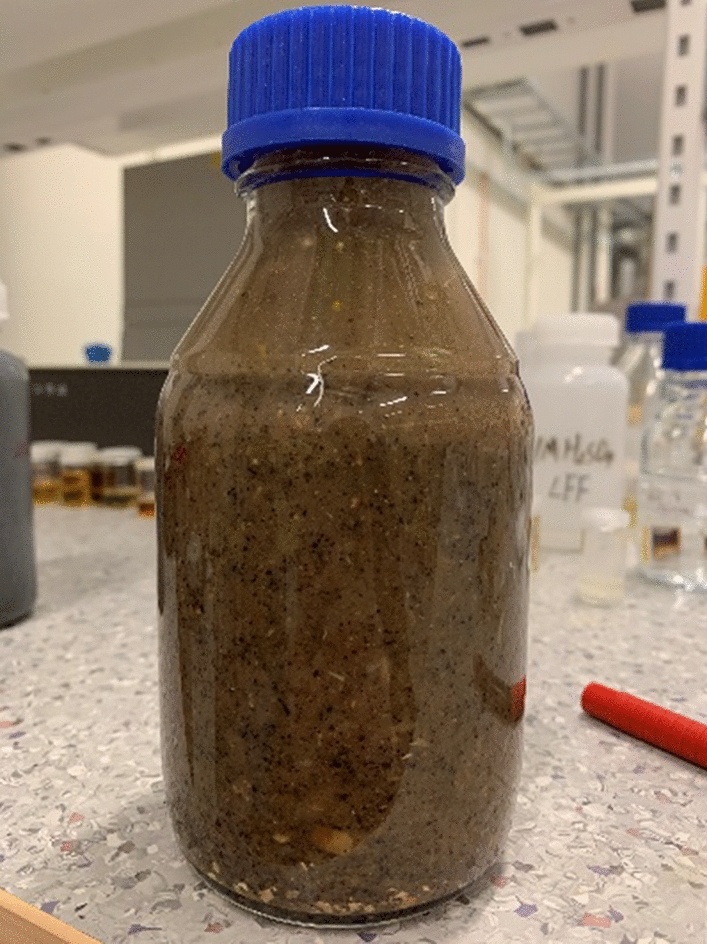
FWS with TS = 10% obtained from Boden Biogas Plant, Sweden

FWS with TS = 10% was sampled from Boden Biogas Plant, Sweden, as depicted in Fig. 9. Its rheological properties were measured using a rheometer (ARES G2, TA Instruments, New Castle, DE, USA) equipped with a helical ribbon impeller in a 34 mm diameter cup located at the Research Institutes of Sweden. The samples were pre-sheared at a shear rate of 50 s^−1^ for 10 s to compensate for any possible sedimentation. Pre-tests were repeated three times using fresh samples at 10 °C, and an average deviation of 11.8% was observed. During testing, shearing was initiated using a low shear rate, and it was increased slowly to avoid sedimentation (*γ* = 100–0.01 s^−1^) at a specific temperature. Rheological properties were tested over a range of temperatures—at 10, 20, 30, 40, 50, 60, and 70 °C. The tested rheology was modeled and used as input for the CFD-based model.

### Numerical modeling of food waste slurry in twisted tubes

In our previous studies [14, 15, 22], the optimal numerical schemes and meshes for CTs and twisted tubes corresponding to slurries obtained from biogas plants were addressed and experimentally validated. Hence, the geometries and boundary conditions adopted in this study are discussed briefly. In addition, the method used for the data reduction of numerical results is also provided below.

#### Geometries and computational domains

**Fig. 10:**
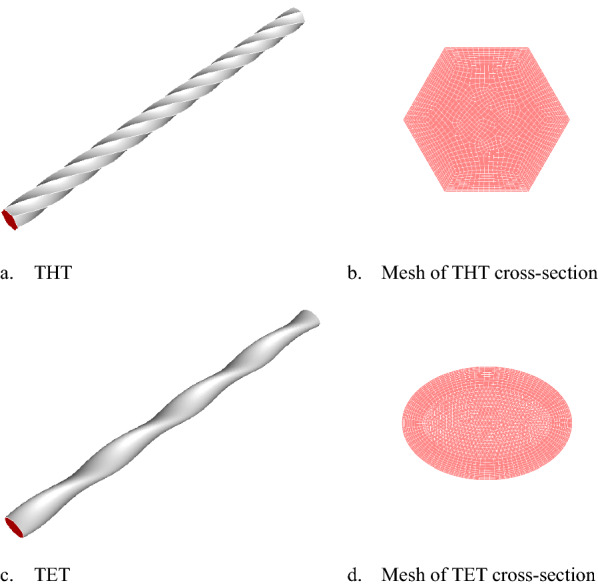
Schematic diagram of the two types of twisted tubes

Figure 10 depicts the geometrical characteristics of THT and TET. The meshes are also illustrated, with cross-sections centered on the centerline. The geometrical parameters of THT, TET, and CT are listed in Table 4. The cross-section at the position, *z* = 0.8 m, in the axial direction was selected to display the details of shear rate distributions in twisted tubes, where the flow is fully developed, stable, and ready for analysis.

**Table 4:** Details of the geometries

	Feature size, m	*d*_*h*_, m	*L*, m	*S*, m	*δ*, mm
THT	*l* = 0.030 m	0.0520	1	0.5	1
TET	*a* = 0.0326 m *b* = 0.0224 m	0.0506	1	0.5	1
CT	–	0.0573	1	–	1

#### Boundary conditions

The flow of the slurry within the twisted tubes and the associated heat-transfer processes were simulated assuming the thermostatic wall condition under constant wall temperature (*T*_*w*_). The specific boundary values of the inlet velocity (*U*) and inlet temperature (*T*_*s*_) are summarized in Table 5. Combinations of these boundary conditions used in specific simulation cases are listed in Additional file 1: Tables S2, S3, and S4 in the additional material. The no-slip boundary condition was assumed in all simulation cases.

**Table 5:** Boundary conditions applied in CFD-based simulation

State	*T*_*s*_, °C	*T*_*w*_, °C	*U*, m/s
Heating	10	60	1.0, 1.5 2.0, 2.25 2.5, 2.75 3.0
	35	60	
Cooling	60	10	
	35	10	

#### Data reduction of numerical results

In the convergent cases, the effective dynamic viscosity (*μ*_*eff*_), inner wall temperature (*T*_*w,i*_), volumetric average of the temperature of FWS (*T*_*s,avg*_), wall-heat flux (*q*), and pressure drop (*ΔP*), were obtained directly from the CFD-based simulations. The heat-transfer coefficient *K*_*i*_ was calculated with Eq. (10). The friction coefficient (*f*) was determined using Eq. (11). Subsequently, the comprehensive parameter, i.e., *PEC* was calculated using Eq. (12) as the enhancement factor for twisted tubes:

10 $$K_{i} = \frac{q}{{T_{w,i} - T_{s,avg} }},$$

11 $$f = \frac{{2d_{h} \Delta P}}{{\rho U^{2} }},$$

12 $$PEC = \frac{{\frac{Nu}{{Nu_{CT} }}}}{{\left( {\frac{f}{{f_{CT} }}} \right)^{{{1 \mathord{\left/ {\vphantom {1 3}} \right. \kern-\nulldelimiterspace} 3}}} }},$$

where heat flux *q*, temperature of inner wall ${T}_{w,i}$, and average temperature of slurry in tubes ${T}_{s,avg}$ are obtained from CFD simulations; the properties of slurry density *ρ*, heat capacity cp, and thermal conductivity λ were calculated according to previous study.

### Pilot-scale testing

**Fig. 11:**
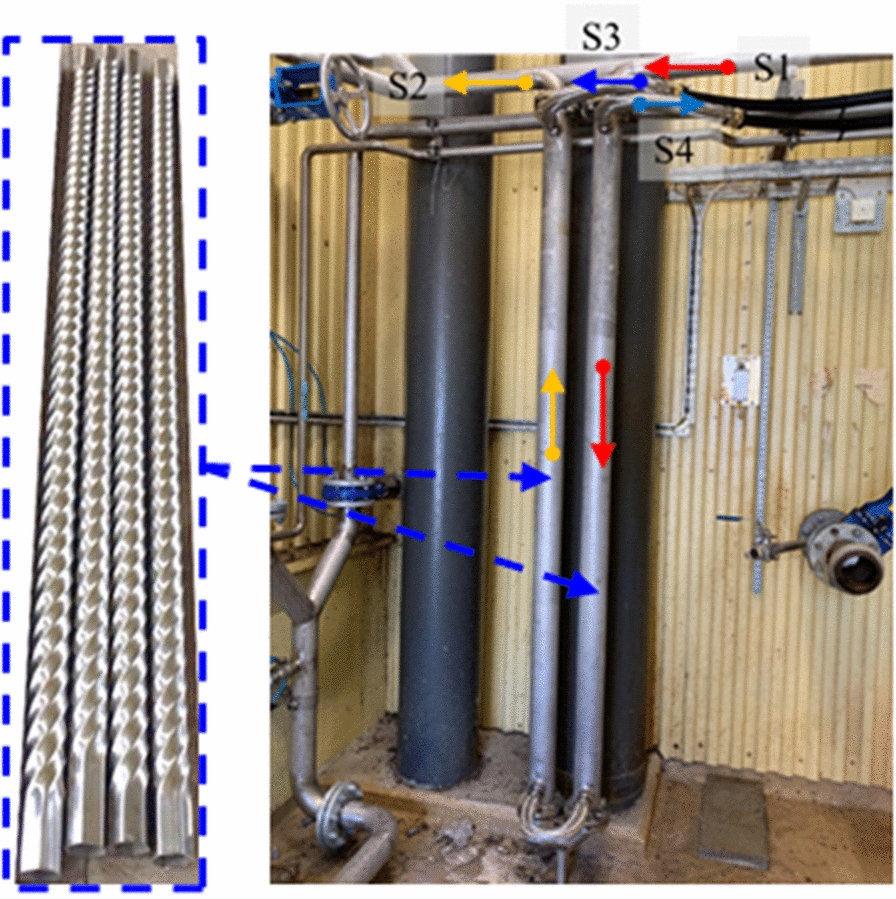
Setup used for pilot test at the Boden Biogas Plant, Sweden; **S1**: inlet of the FWS (tube side, 55 °C, 5 m^3^/h); **S2**: outlet of the FWS; **S3**: inlet of cold water (shell side, 10 °C, 10 m^3^/h); **S4**: outlet of cold water

THTs were manufactured and installed as a type of shell-and-tube heat exchanger at the Boden Biogas Plant, Sweden. Their geometric parameters were taken to be identical to those of the “model”, as listed in Table 4. The diameter of the shell side was 0.08 m and the total length of the heat exchangers was 4.4 m. As depicted in Fig. 11, the working fluid was taken to be FWS with TS = 10%. The inlet volume flow rate and temperature of the FWS on the tube side were maintained at 10 m^3^/h and 55 °C, respectively. The inlet volume flow rate and temperature of the cooling water on the shell side were maintained at 10 m^3^/h and 10 °C, respectively. The outlet temperatures on both the tube and shell sides were recorded using Pt-100 temperature sensors. The heat-transfer coefficient (*K*_*i*_), of THT was calculated using the method described in our previous study [15].

## Supplementary Information

**Additional file 1**:** Table S1.** Tested data of rheological properties for FWS with TS = 10%. **Table S2.** Numerical results of FWS in THT. **Table S3**. Numerical results of FWS in TET. **Table S4.** Numerical results of FWS in CT

## Data Availability

The data supporting the conclusions of this article are included with the article and its supplementary material.

## Abbreviations

AD: Anaerobic digestion
FWS: Food waste slurry
TS: Total solid
THT: Twisted hexagonal tube
CFD: Computational fluid dynamics
MS: Manure slurry
CSS: Corn straw slurry
TET: Twisted elliptical tube
CT: Circular tube
CSV: Critical-shear viscosity
ZSV: Zero-shear viscosity
ARD: Average relative deviation
*PEC*: Performance evaluation criteria

## List of symbols

*Re*: Reynolds number
*Nu*: Nusselt number
*Pr*: Prandtl number
*k*: Consistency coefficient
*n*: Flow behavior index
*K*_*i*_: Heat-transfer coefficient, W/(m^2^·K)
*T*: Temperature, °C
*ΔT*: Temperature difference of wall and fluid, °C
*ΔP*: Pressure drop, Pa
*f*: Friction coefficient
*U*: Velocity, m/s
*d*_*h*_: Hydraulic diameter, m
*L*: Length of the tubes, m
*S*: Torque of tubes, m
*c*_*p*_: Heat capacity, J/(kg·K)
*q*: Heat flux, W/m^2^

## Greek alphabets

*γ*: Shear rate, s^−^^1^
*μ*: Dynamic viscosity, Pa·s
*δ*: Thickness of tubes, m
*ρ*: Density, kg/m^3^
*λ*: Thermal conductivity of slurry, W/(m·K)

## Subscripts

*s*: Slurry
*w*: Wall
*eff*: Effective
*avg*: Average
*bl*: Boundary layer
*i*: Inner
