# Supplementary material for: Heat-transfer performance of twisted tubes for highly viscous food waste slurry from biogas plants
Source: Biotechnol Biofuels Bioprod. 2022 Jul 6;15:74. doi: 10.1186/s13068-022-02156-4 (PMC9261055; doi:10.1186/s13068-022-02156-4)
Supplement: Supplementary file 1 — Additional file 1: Table S1. Tested data of rheological properties for FWS with TS = 10%. Table S2. Numerical results of FWS in THT. Table S3. Numerical results of FWS in TET. Table S4. Numerical results of FWS in CT [file 13068_2022_2156_MOESM1_ESM.docx]

**Additional file 1 material**

Table. S1 Tested data of rheological properties for FWS with TS = 10%

| Shear rate *γ*  s^-1^ | Dynamic viscosity *μ* (Pa·s) | | | | | | |
| --- | --- | --- | --- | --- | --- | --- | --- |
|  | 10 °C | 20 °C | 30 °C | 40 °C | 50 °C | 60 °C | 70 °C |
| 100.0 | 0.87 | 0.65 | 0.51 | 0.41 | 0.37 | 0.29 | 0.25 |
| 63.1 | 1.36 | 0.90 | 0.71 | 0.62 | 0.53 | 0.39 | 0.36 |
| 39.8 | 1.99 | 1.25 | 1.00 | 0.88 | 0.74 | 0.55 | 0.51 |
| 25.1 | 2.87 | 1.85 | 1.59 | 1.30 | 1.05 | 0.88 | 0.82 |
| 15.9 | 4.02 | 2.89 | 2.17 | 1.78 | 1.49 | 1.31 | 1.25 |
| 10.0 | 6.16 | 3.89 | 2.84 | 2.84 | 2.26 | 2.39 | 1.88 |
| 6.31 | 9.10 | 6.85 | 4.17 | 4.52 | 3.98 | 3.11 | 2.95 |
| 3.98 | 14.48 | 11.3 | 8.06 | 5.63 | 5.59 | 4.80 | 4.02 |
| 2.51 | 25.20 | 15.0 | 13.5 | 11.7 | 10.2 | 9.52 | 7.25 |
| 1.59 | 34.67 | 25.9 | 21.2 | 17.0 | 14.1 | 13.1 | 9.40 |
| 1.00 | 57.63 | 42.6 | 36.0 | 30.2 | 22.7 | 20.5 | 20.1 |
| 0.63 | 99.87 | 69.7 | 55.5 | 48.2 | 44.1 | 39.7 | 35.4 |
| 0.40 | 132.7 | 102.4 | 86.8 | 77.0 | 63.1 | 58.9 | 50.2 |
| 0.25 | 225.3 | 170.6 | 136.2 | 123.1 | 117.0 | 112.9 | 101.2 |
| 0.16 | 361.6 | 251.0 | 217.7 | 189.0 | 173.0 | 145.4 | 136.6 |
| 0.10 | 691.9 | 484.8 | 376.3 | 345.2 | 307.7 | 266.4 | 241.0 |
| 0.063 | 1024.1 | 738.4 | 582.5 | 554.5 | 484.3 | 441.4 | 415.1 |
| 0.040 | 1530.6 | 1150.5 | 943.2 | 872.4 | 869.4 | 819.7 | 664.3 |
| 0.025 | 2278.1 | 1841.6 | 1626.2 | 1319.6 | 1250.6 | 1191.2 | 1053.9 |
| 0.016 | 3599.4 | 2829.3 | 2626.6 | 2106.9 | 1966.8 | 1887.4 | 1665.0 |
| 0.010 | 5604.8 | 5012.1 | 4183.4 | 3457.0 | 3213.0 | 3180.6 | 2643.6 |

Table. S2 Numerical results of FWS in THT

| Input parameter | | | Numerical results | | | | | | | | |
| --- | --- | --- | --- | --- | --- | --- | --- | --- | --- | --- | --- |
| *T_w_*  °C | *T_s_*  °C | *U*  m/s | *γ_bl_*  s^-1^ | *μ_eff_*  Pa·s | *T_s,avg_*  °C | *T_w,i_*  °C | *K_i_*  W/(m^2^·K) | *Re* | *Pr* | *Nu* | *f* |
| 10 | 60 | 1 | 88.4 | 16.4 | 53.4 | 11.0 | 367 | 3.16 | 111615 | 30.9 | 0.250 |
| 10 | 60 | 1.5 | 128.4 | 14.8 | 54.6 | 11.7 | 607 | 5.29 | 100180 | 51.1 | 0.127 |
| 10 | 60 | 2 | 161.1 | 17.2 | 56.8 | 13.4 | 1165 | 6.04 | 116883 | 98.1 | 0.079 |
| 10 | 60 | 2.25 | 178.4 | 12.1 | 57.8 | 14.5 | 1585 | 9.66 | 82295 | 133.5 | 0.049 |
| 10 | 60 | 2.5 | 186.8 | 13.4 | 58.9 | 18.0 | 2939 | 9.73 | 90698 | 247.6 | 0.049 |
| 10 | 60 | 2.75 | 191.9 | 19.1 | 59.2 | 20.5 | 4091 | 7.50 | 129421 | 344.7 | 0.060 |
| 10 | 60 | 3 | 200.9 | 16.6 | 59.2 | 21.6 | 4669 | 9.42 | 112460 | 393.5 | 0.056 |
| 10 | 35 | 2 | 161.7 | 13.6 | 34.4 | 12.7 | 1893 | 7.68 | 91983 | 159.5 | 0.073 |
| 10 | 35 | 2.25 | 180.3 | 16.3 | 34.5 | 12.9 | 2062 | 7.17 | 110846 | 173.8 | 0.061 |
| 10 | 35 | 2.75 | 215.1 | 25.7 | 34.6 | 13.7 | 2669 | 5.56 | 174597 | 224.9 | 0.062 |
| 10 | 35 | 3 | 231.9 | 21.5 | 34.7 | 14.3 | 3155 | 7.26 | 145870 | 265.9 | 0.054 |
| 60 | 10 | 1 | 79.8 | 35.2 | 11.2 | 54.0 | 2099 | 1.48 | 238982 | 176.9 | 0.422 |
| 60 | 10 | 1.5 | 119.8 | 32.9 | 11.0 | 54.1 | 2062 | 2.38 | 222962 | 173.8 | 0.184 |
| 60 | 10 | 2 | 158.9 | 32.4 | 10.7 | 53.1 | 2447 | 3.21 | 219730 | 206.2 | 0.132 |
| 60 | 10 | 2.25 | 179.8 | 20.7 | 10.9 | 53.5 | 2299 | 5.66 | 140324 | 193.7 | 0.081 |
| 60 | 10 | 2.5 | 199.1 | 25.5 | 10.9 | 53.2 | 2407 | 5.11 | 172940 | 202.8 | 0.074 |
| 60 | 10 | 2.75 | 218.5 | 26.7 | 10.8 | 53.0 | 2525 | 5.36 | 181164 | 212.7 | 0.069 |
| 60 | 10 | 3 | 238.3 | 25.3 | 10.8 | 52.8 | 2611 | 6.16 | 172016 | 220.0 | 0.060 |
| 60 | 35 | 2 | 157.8 | 35.4 | 35.4 | 56.5 | 2535 | 2.94 | 240177 | 213.7 | 0.084 |
| 60 | 35 | 2.25 | 177.3 | 33.1 | 35.4 | 56.3 | 2657 | 3.53 | 224936 | 223.9 | 0.070 |
| 60 | 35 | 2.75 | 214.4 | 29.8 | 35.3 | 55.9 | 3036 | 4.80 | 202346 | 255.9 | 0.062 |
| 60 | 35 | 3 | 229.6 | 26.1 | 35.3 | 55.5 | 3377 | 5.99 | 177005 | 284.6 | 0.056 |

Table. S3 Numerical results of FWS in TET

| Input parameter | | | Numerical results | | | | | | | | |
| --- | --- | --- | --- | --- | --- | --- | --- | --- | --- | --- | --- |
| *T_w_*  °C | *T_s_*  °C | *U*  m/s | *γ_bl_*  s^-1^ | *μ_eff_*  Pa·s | *T_s,avg_*  °C | *T_w,i_*  °C | *K_i_*  W/(m^2^·K) | *Re* | *Pr* | *Nu* | *f* |
| 10 | 60 | 1 | 87.2 | 5.0 | 57.5 | 13.3 | 1126 | 10.16 | 33818 | 92.3 | 0.313 |
| 10 | 60 | 1.5 | 129.9 | 3.0 | 58.1 | 14.4 | 1508 | 25.25 | 20417 | 123.6 | 0.135 |
| 10 | 60 | 2 | 177.9 | 2.4 | 59.0 | 15.7 | 1972 | 41.84 | 16426 | 161.7 | 0.065 |
| 10 | 60 | 2.25 | 202.6 | 1.9 | 59.1 | 15.9 | 2044 | 61.52 | 12568 | 167.6 | 0.043 |
| 10 | 60 | 2.5 | 225.1 | 2.5 | 59.3 | 16.2 | 2186 | 50.88 | 16887 | 179.3 | 0.041 |
| 10 | 60 | 2.75 | 249.9 | 2.0 | 59.4 | 16.4 | 2255 | 68.92 | 13713 | 184.9 | 0.035 |
| 10 | 60 | 3 | 275.0 | 2.7 | 59.5 | 16.6 | 2311 | 55.29 | 18647 | 189.5 | 0.029 |
| 10 | 35 | 2 | 174.5 | 3.3 | 34.5 | 12.8 | 1923 | 30.37 | 22629 | 157.7 | 0.107 |
| 10 | 35 | 2.25 | 197.9 | 2.5 | 34.5 | 12.9 | 2002 | 44.93 | 17208 | 164.2 | 0.077 |
| 60 | 35 | 2.5 | 219.1 | 2.7 | 34.6 | 13.0 | 2106 | 46.83 | 18347 | 172.7 | 0.070 |
| 10 | 35 | 2.75 | 242.8 | 2.4 | 34.7 | 13.1 | 2181 | 58.03 | 16286 | 178.8 | 0.059 |
| 10 | 35 | 3 | 267.2 | 2.6 | 34.7 | 13.2 | 2234 | 59.11 | 17441 | 183.2 | 0.051 |
| 60 | 10 | 1 | 84.0 | 10.0 | 11.2 | 54.8 | 1843 | 5.04 | 68132 | 151.1 | 0.415 |
| 60 | 10 | 1.5 | 126.3 | 8.0 | 10.8 | 54.3 | 2017 | 9.52 | 54158 | 165.4 | 0.217 |
| 60 | 10 | 2 | 168.4 | 5.7 | 10.7 | 54.0 | 2106 | 17.67 | 38898 | 172.7 | 0.131 |
| 60 | 10 | 2.25 | 189.9 | 3.9 | 10.6 | 53.9 | 2135 | 29.34 | 26350 | 175.1 | 0.104 |
| 60 | 10 | 2.5 | 211.5 | 3.5 | 10.6 | 53.8 | 2163 | 36.39 | 23611 | 177.4 | 0.088 |
| 60 | 10 | 2.75 | 233.1 | 3.2 | 10.5 | 53.7 | 2188 | 43.84 | 21555 | 179.4 | 0.077 |
| 60 | 10 | 3 | 255.1 | 2.6 | 10.5 | 53.7 | 2208 | 58.31 | 17682 | 181.1 | 0.067 |
| 60 | 35 | 2 | 169.1 | 3.5 | 35.3 | 57.0 | 2097 | 28.74 | 23916 | 171.9 | 0.080 |
| 60 | 35 | 2.25 | 191.0 | 2.4 | 35.3 | 57.0 | 2128 | 46.84 | 16509 | 174.5 | 0.065 |
| 10 | 35 | 2.5 | 214.3 | 2.3 | 35.3 | 56.9 | 2198 | 55.73 | 15417 | 180.2 | 0.060 |
| 60 | 35 | 2.75 | 237.7 | 1.8 | 35.3 | 56.8 | 2248 | 75.30 | 12551 | 184.4 | 0.055 |
| 60 | 35 | 3 | 262.2 | 1.6 | 35.3 | 56.7 | 2288 | 92.81 | 11109 | 187.6 | 0.049 |

Table. S4 Numerical results of FWS in CT

| Input parameter | | | Numerical results | | | | | | | | |
| --- | --- | --- | --- | --- | --- | --- | --- | --- | --- | --- | --- |
| *T_w_*  °C | *T_s_*  °C | *U*  m/s | *γ_bl_*  s^-1^ | *μ_eff_*  Pa·s | *T_s,avg_*  °C | *T_w,i_*  °C | *K_i_*  W/(m^2^·K) | *Re* | *Pr* | *Nu* | *f* |
| 10 | 60 | 1 | 75.0 | 43.8 | 57.0 | 11.6 | 428 | 1.31 | 297270 | 49.0 | 0.549 |
| 10 | 60 | 1.5 | 115.5 | 19.5 | 56.0 | 11.3 | 527 | 4.41 | 132525 | 39.8 | 0.227 |
| 10 | 60 | 2 | 149.5 | 23.7 | 57.5 | 12.3 | 781 | 4.84 | 160877 | 72.6 | 0.104 |
| 10 | 60 | 2.25 | 167.0 | 29.0 | 57.8 | 12.6 | 863 | 4.45 | 196821 | 80.2 | 0.084 |
| 10 | 60 | 2.5 | 182.7 | 35.6 | 58.3 | 13.0 | 998 | 4.03 | 241815 | 92.7 | 0.086 |
| 10 | 60 | 2.75 | 201.7 | 28.9 | 58.2 | 13.1 | 1044 | 5.46 | 196225 | 97.0 | 0.060 |
| 10 | 60 | 3 | 219.2 | 30.1 | 58.5 | 13.5 | 1159 | 5.72 | 204109 | 107.7 | 0.042 |
| 10 | 35 | 2 | 149.9 | 32.1 | 33.7 | 11.2 | 780 | 3.58 | 217536 | 72.5 | 0.137 |
| 10 | 35 | 2.25 | 167.8 | 34.3 | 33.9 | 11.3 | 863 | 3.76 | 233089 | 80.2 | 0.109 |
| 60 | 35 | 2.5 | 183.8 | 41.5 | 34.1 | 11.5 | 979 | 3.46 | 281664 | 91.0 | 0.102 |
| 10 | 35 | 2.75 | 202.3 | 36.1 | 34.1 | 11.6 | 1038 | 4.37 | 245286 | 96.4 | 0.079 |
| 10 | 35 | 3 | 219.7 | 36.4 | 34.2 | 11.7 | 1128 | 4.73 | 247048 | 104.8 | 0.067 |
| 60 | 10 | 1 | 71.0 | 54.3 | 11.0 | 56.4 | 1212 | 1.06 | 368260 | 112.6 | 0.227 |
| 60 | 10 | 1.5 | 108.0 | 43.2 | 10.8 | 55.8 | 1405 | 1.99 | 293196 | 130.6 | 0.127 |
| 60 | 10 | 2 | 142.1 | 51.6 | 10.7 | 55.4 | 1546 | 2.23 | 349917 | 143.6 | 0.075 |
| 60 | 10 | 2.25 | 159.4 | 57.2 | 10.6 | 55.3 | 1595 | 2.26 | 388023 | 148.2 | 0.059 |
| 60 | 10 | 2.5 | 177.1 | 57.3 | 10.6 | 55.1 | 1652 | 2.50 | 389063 | 153.5 | 0.050 |
| 60 | 10 | 2.75 | 194.9 | 55.4 | 10.5 | 55.0 | 1692 | 2.85 | 375989 | 157.2 | 0.043 |
| 60 | 10 | 3 | 212.6 | 55.6 | 10.5 | 54.9 | 1733 | 3.09 | 377515 | 161.0 | 0.037 |
| 60 | 35 | 2 | 142.1 | 47.8 | 35.4 | 57.8 | 1497 | 2.40 | 324482 | 139.1 | 0.046 |
| 60 | 35 | 2.25 | 159.6 | 55.5 | 35.3 | 57.7 | 1554 | 2.33 | 376701 | 144.4 | 0.033 |
| 10 | 35 | 2.5 | 177.3 | 56.6 | 35.3 | 57.6 | 1601 | 2.53 | 384064 | 148.7 | 0.029 |
| 60 | 35 | 2.75 | 195.0 | 54.9 | 35.3 | 57.6 | 1653 | 2.87 | 372851 | 153.6 | 0.023 |
| 60 | 35 | 3 | 212.7 | 55.3 | 35.3 | 57.5 | 1697 | 3.11 | 375263 | 157.7 | 0.020 |
